# Effect of Schmidt number on mass transfer across a sheared gas-liquid interface in a wind-driven turbulence

**DOI:** 10.1038/srep37059

**Published:** 2016-11-14

**Authors:** Naohisa Takagaki, Ryoichi Kurose, Atsushi Kimura, Satoru Komori

**Affiliations:** 1 Department of Mechanical Engineering, University of Hyogo, Himeji 671-2280, Japan; 2 Department of Mechanical Engineering and Science, Kyoto University, Kyoto 615-8540, Japan; 3 Research Center for Highly-Functional Nanoparticles, Doshisha University, Kyotanabe 610-0394, Japan; 4 Center for Earth Information Science and Technology (CEIST), Japan Agency for Marine-Earth Science and Technology (JAMSTEC), Yokohama, 236-0001, Japan

## Abstract

The mass transfer across a sheared gas-liquid interface strongly depends on the Schmidt number. Here we investigate the relationship between mass transfer coefficient on the liquid side, *k*_L_, and Schmidt number, *Sc*, in the wide range of 0.7 ≤ *Sc* ≤ 1000. We apply a three-dimensional semi direct numerical simulation (SEMI-DNS), in which the mass transfer is solved based on an approximated deconvolution model (ADM) scheme, to wind-driven turbulence with mass transfer across a sheared wind-driven wavy gas-liquid interface. In order to capture the deforming gas-liquid interface, an arbitrary Lagrangian-Eulerian (ALE) method is employed. Our results show that similar to the case for flat gas-liquid interfaces, *k*_L_ for the wind-driven wavy gas-liquid interface is generally proportional to *Sc*^−0.5^, and can be roughly estimated by the surface divergence model. This trend is endorsed by the fact that the mass transfer across the gas-liquid interface is controlled mainly by streamwise vortices on the liquid side even for the wind-driven turbulence under the conditions of low wind velocities without wave breaking.

Mass transfer phenomena across gas-liquid interfaces are often seen in geophysical and industrial processes, and such mass transfer is believed to be enhanced by wind-driven turbulence (i.e., surface-renewal motions) near the interface on the liquid side (e.g., Jähne *et al*.^1^,^2^,^3^, Komori *et al*.^4^,^5^, Takagaki *et al*.^6^, Kurose *et al*.^7^). In order to clarify the mass transfer mechanism and precisely evaluate the amount of the mass transferred across the wind-driven wavy gas-liquid interface, direct numerical simulations (DNSs) of gas-liquid two-phase turbulent flows with wind-driven wavy interfaces were carried out by some researchers (Komori *et al*.^5^, Takagaki *et al*.^6^, Kunugi *et al*.^8^, Lakehal *et al*.^9^,^10^,^11^, Banerjee^12^, Banerjee *et al*.^13^). One of the most important properties to globally predict the amount of mass transferred across such gas-liquid interfaces is the mass transfer coefficient, *k*_L_, and therefore a precise model for it is necessary. Here, *k*_L_ is defined as:


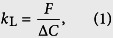


where *F* is the mass flux at the gas-liquid interface per unit area, Δ*C* the mass concentration difference between the interface and the bulk liquid. The value of *k*_L_ is often correlated with the Schmidt number, *Sc* (=*ν*_L_/*D*_L_), on the liquid side, where *ν*_L_ and *D*_L_ are the kinematic viscosity and molecular diffusivity on the liquid side, respectively. However, the evaluations of *k*_L_ are limited for *Sc* ≤ 1600 in previous experiments (e.g., Jähne *et al*.^1^,^2^,^3^, Komori *et al*.^4^, Liss^14^, Broecker *et al*.^15^, Wanninkhof^16^, Iwano *et al*.^17^,^18^) and limited for *Sc* ≤ 100 in previous numerical simulations (e.g., Komori *et al*.^5^, Takagaki *et al*.^6^, Banerjee *et al*.^13^). Also, since the flow and mass conditions are different among these previous experimental and numerical studies, the universal relation between *k*_L_ and *Sc* has not been clarified yet, even in these *Sc* ranges.

In this paper, we aim to present the relationship between *k*_L_ and *Sc* in the wide range of 0.7 ≤ *Sc* ≤ 1000 by applying a SEMI-DNS, in which the mass transfer is solved based on an approximated deconvolution model (ADM) scheme proposed by Stolz and Adams^19^, to a gas-liquid two-phase turbulent flow with a wind-driven wavy interface.

## Methods

The numerical procedure of the DNS used here was the same as in the study by Komori *et al*.^5^ and Takagaki *et al*.^6^. In the procedure, the wind-driven wavy gas-liquid interface was captured by the arbitrary Lagrangian-Eulerian (ALE) method with boundary-fitted coordinates (BFC) on moving grids (Komori *et al*.^5^,^20^, Takagaki *et al*.^6^, Lakehal *et al*.^9^,^10^,^11^, Banerjee *et al*.^13^, Fulgosi *et al*.^21^, Lin *et al*.^22^, Guo and Shen^23^, Tsai *et al*.^24^). The non-dimensional governing equations for an incompressible Newtonian fluid flow with mass transfer are given by the equation of continuity, Navier-Stokes (N-S) equation, and transport equations of mass using the Einstein summation convention:


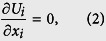



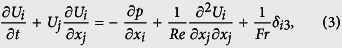



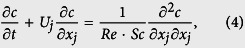


where *U*_*i*_ is the *i* th component of the velocity vector (*i* = 1, 2, and 3 denote the streamwise, spanwise and vertical directions, respectively), *p* the pressure, *δ*_*ij*_ the Kronecker’s delta, and *c* the mass concentration. Here, equation (4) is solved only on the liquid side. The non-dimensional parameters, Reynolds number *Re*, Schmidt number *Sc* and Froude number *Fr* are defined as:


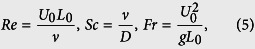


where *L*_0_ and *U*_0_ are the reference length and velocity, respectively, *ν* the kinematic viscosity, *D*_*L*_ the molecular diffusivity of mass on the liquid side, and *g* the acceleration due to gravity. On the gas-liquid interface, two boundary conditions should be satisfied. One is the kinematic boundary condition that describes the Lagrangian behavior of the fluid particle on the mobile gas-liquid interface, and the other is the dynamic boundary condition, which is determined from the balance of stresses acting on the gas-liquid interface in the normal and tangential directions (Komori *et al*.^5^, Takagaki *et al*.^6^).

Since a high *Sc* mass causes a smaller Batchelor scale (Hasegawa and Kasagi^25^,^26^,^27^, Kurose *et al*.^28^,^29^), the mass transfer was solved based on ADM scheme proposed by Stolz and Adams^19^ and modified by Mathew *et al*.^30^. The model based on DNS of flow field and ADM of mass field is proposed by Schwertfirm and Manhart^31^ and is called as SEMI-DNS. The ADM method is briefly explained here, and see more details in Schwertfirm and Manhart^31^. When a mass concentration field, *f*, is predicted with meshes coarser than the Batchelor scale, the mass concentration field is like a filtered mass concentration field, 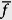. Now, by the filtering function, *G*, the relation between *f* and 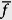 are 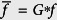, where * is the filtering procedure. On the ADM method, without any LES model, the mass concentration field is predicted using the inverse filtering function, *Q*. Generally, under the assumption of the existence of the inverse matrix, *G*^−1^, the Taylor expansion of *G*^−1^ is shown as:


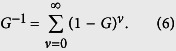


By equation (6), Stolz and Adams^19^ defines *Q* as:


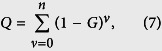


and, therefore, *Q* has the property of inverse matrix, that is, Q*G = G*Q ~ *I* and *Q* ~ *G*^−1^. Therefore, 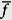 can be restored by *Q* shown as:


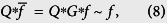


where they defines 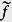 as 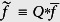. In this study, the simple filtering shown as:


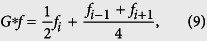


is used. Uisng equation (8), equation (4) is rewritten (see the details in Schwertfirm and Manhart^31^) as:


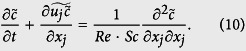


**Figure 1:**
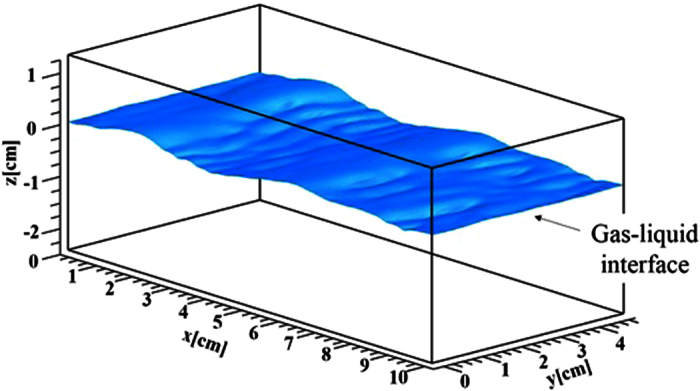
Instantaneous configuration of wind-driven wavy gas-liquid interface at *t* = 5.0 s.

The computational domain was 8*δ* × 3.92*δ* × 3*δ (δ* = 1.25 × 10^−2^ m) in the streamwise (*x*), spanwise (*y*) and vertical (*z*) directions. The origin (*x* = *y* = *z* = 0) was located at the height of 2*δ* from the bottom, and the initial flat gas-liquid interface which divides the two-phase flow between upper gas and lower liquid streams was placed on the plane of *z* = 0. The grid points used in the streamwise (*x*), spanwise (*y*) and vertical (*z*) directions were 200 × 98 × 60 on the gas side and 200 × 98 × 120 on the liquid side, respectively. The grid spacing was equidistant in the streamwise (*x*) and spanwise (*y*) directions, and to get high resolution the nonuniform meshes clustered in the gas-liquid-interface region were used in the vertical (*z*) direction. Periodic boundary conditions were applied in the streamwise (*x*) and spanwise (*y*) directions, and slip boundary conditions were applied at the top and bottom boundaries. As initial conditions of flow field, a fully developed wall turbulent flow and a quiescent flow were given on the gas and liquid sides of the initial flat interface, respectively. For the computation of the mass transfer on the liquid side, the boundary conditions of the passive mass at the gas-liquid interface and the bottom boundary were given by *C* = 1.0 and Neumann condition, respectively. The marker and cell (MAC) (Harlow and Welch^32^) method was used to solve the Navier-Stokes equations. In order to induce the realistic deformation of the gas-liquid interface, a fully developed wall turbulence for an initial free stream wind speed of *U*_∞,ini_ = 4.8 m/s and an initial friction velocity of u_*,ini_ = 0.242 m/s was given on the gas side over a flat quiescent liquid. The gas flow was driven by pressure gradient imposed in the streamwise direction. The Reynolds number based on *U*_∞,ini_ and *δ, Re*_ini_, was 3960 and that based on u_*,ini_ and δ, Re^*^_ini_, was 200. The density ratio of the gas and liquid was 830, which corresponds to the value for the air-water two phase flow at about 20 °C. The Schmidt number, *Sc*, on the liquid side ranged from 0.7 to 1000 (11 cases). For comparison, the computations for a forced flat gas-liquid interface were also carried out in the same conditions as for the wind-driven wavy gas-liquid interface (11 cases). Each computation was performed until the flow field on the liquid side reaches statistically steady state. The CPU times for the wind-driven wavy and flat interfaces were about 3900 hours (10 days of wall-clock time using 16 cores) and 5200 hours (14 days of wall-clock time using 16 cores) for 2,400,000 steps and 6,400,000 steps (6.0 seconds and 16.0 seconds) on the super computer NEC:SX-ACE, respectively. Figure 1 shows the instantaneous configuration of the wind-driven gas-liquid interface at a fully-developed time.

## Results and Discussion

**Figure 2:**
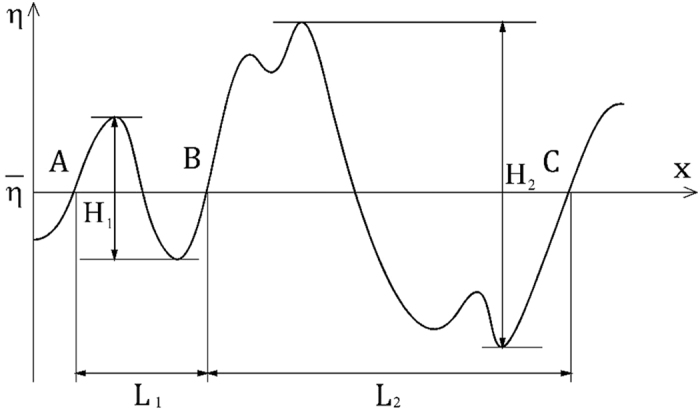
Sketch of zero-up cross method.

The mass transfer coefficient on the liquid side, *k*_L_, (Eq. (1)) is calculated as:


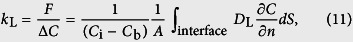


where Δ*C* = *C*_i_ − *C*_b_ (here, *C*_i_ and *C*_b_ are the mass concentrations of the interface and bulk liquid, respectively, and *C*_b_ is set to be zero in this study), *n* the normal direction with respect to the gas-liquid interface, *A* the surface area of the interface, and *D*_L_ the molecular diffusivity of the mass on the liquid side. For both the wind-driven wavy and flat interfaces, the statistics were taken after the flows fully develop and the values of *k*_L_ indicate statistically steady values. That is, in the wind-driven wavy interface, we started the wind-water simulation with initial flat air-water interface, defined as *t* = 0 s. We started to solve the mass concentration field also at *t* = 0 s. We calculated a time-averaged *k*_L_ by use of the the mass concentration field during the time period *t* = 5.0 s to 6.0 s. In flat interface condition, we similarly started the wind-water simulation at *t* = 0 s, but we did not start to solve the mass concentration field until *t* = 10 s, and we calculated a time-averaged *k*_L_ by use of the mass concentration field from *t* = 15.0 s to 16.0 s. The statistics of wind and waves are also taken in the same manner as Komori *et al*.^5^ and Takagaki *et al*.^6^, and the values are listed in Table 1. Here, the uniform velocity on the gas side, *U*_∞_, is defined as the velocity on the upper wall of the computational domain. The wind speed at 10 m height above the gas-liquid interface, *U*_10N_, drag coefficient, *C*_DN_, and surface current, *U*_SURF_, are estimated in the same manner as Komori *et al*.^5^ and Takagaki *et al*.^6^. Each wind wave is determined by applying the zero-up cross method to the spatial fluctuation of the water level. First of all, we obtained the streamwise distribution of surface elevation (Fig. 2). Using the zero-up cross method, we detected locations with zero-up cross (see positions A, B, and C in Fig. 2). Then, for example, a wave is defined as being in the area between positions of A and B in Fig. 2. From those waves, we selected the largest one-third waves, and defined the significant waves as the largest one-third waves. The significant wave height, *H*_S_, and significant wave length, *L*_S_, are defined as the mean wave height and length, respectively, for the largest one-third waves. The phase speed of the significant wind-waves, *C*_p_, is measured by analyzing the propagation of the significant wind-waves.

**Figure 3:**
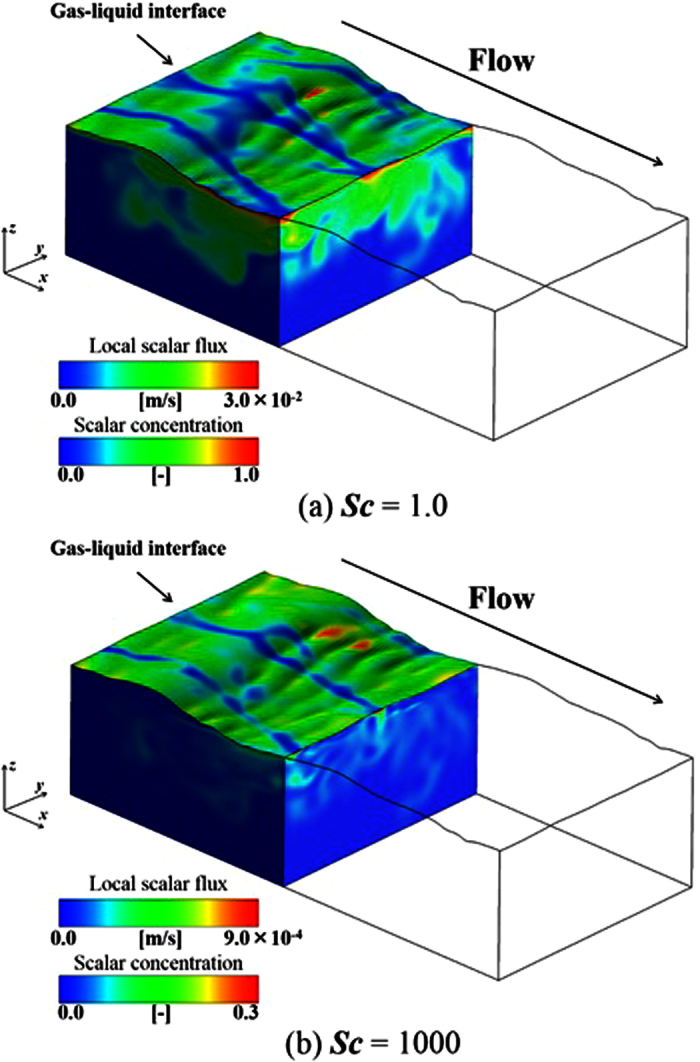
Relationship between distributions of instantaneous local mass flux on the gas–liquid interface, *F*_local_, and mass concentration on the liquid side (*y-z* and *z-x* planes) for the wind-driven wavy gas-liquid interface at *t* = 5.0 s for (**a**) *Sc* = 1.0 and (**b**) *Sc* = 1000. Only liquid sides are shown. It should be noted that the color ranges of *F*_local_ and mass concentration for *Sc* = 1.0 and 1000 are different.

Figure 3 shows the relationship between distributions of instantaneous local mass flux on the gas-liquid interface and the mass concentration on the liquid side (*y-z* and *z-x* planes) for the wind-driven wavy interface for *Sc* = 1.0 and 1000 at *t* = 5.0 s. Here, the local mass flux, *F*_local_, on the gas-liquid interface is estimated by:


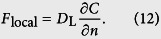


It should be noted that the color ranges of *F*_local_ and the mass concentration for *Sc* = 1.0 and 1000 are different in this figure. The distributions of *F*_local_ and the mass concentration on the liquid side for *Sc* = 1.0 and 1000 are observed to be similar, namely, streaky motions of the mass flux on the gas-liquid interface are strongly associated with the streamwise vortices related to downward bursting motions appearing beneath the interface. The mass transfer mechanism across the wind-driven wavy interface is illustrated in Figure 24 in Komori *et al*.^5^. In summary, a pair of streamwise vortices causes downward bursting motions beneath the streaky regions with both low-mass flux and high streamwise velocity of the gas-liquid interface, and the peeling process happens between the downward bursting motions. Due to this process, the surface layer thickness is reduced and the gradient of the mass concentration increases, and then the mass flux is enhanced. It is evident that the distribution of *F*_local_ is streaky and the amount of the mass entrained into the liquid side is lower for *Sc* = 1000 than those for *Sc* = 1.0 due to the lower diffusivity, although the locations of the low-*F*_local_ streaks on the gas-liquid interface are identical between them. It is also confirmed, from the figure, that high *Sc* mass (*Sc* = 1000) induces a smaller scale structure than low *Sc* mass (*Sc* = 1.0).

**Figure 4:**
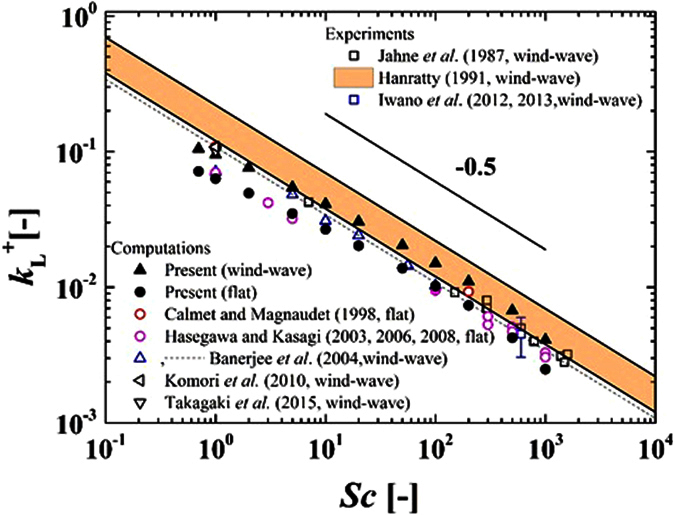
Relationship between *k*_L_^+^ and *Sc* together with previous studies.

Figure 4 shows the relationship between the mass transfer coefficient, *k*_L_^+^, normalized by the friction velocity on the liquid side and *Sc*, together with those obtained by previous experiments (Jähne *et al*.^3^, Iwano *et al*.^17^,^18^, Hanratty^33^) and computations for the wind-driven wavy interface (Komori *et al*.^5^, Takagaki *et al*.^6^, Banerjee *et al*.^13^) and the flat interface (Hasegawa and Kasagi^27^,^34^,^35^, Calmet and Magnaudet^36^). Here, Hanratty’s^33^ range is obtained using the experimental data of Jähne *et al*.^2^, Liss^14^, Broecker *et al*.^15^, and Merlivat and Memery^37^. Similar to the previous studies, the predicted *k*_L_^+^ for both the wind-driven wavy and flat interfaces tend to be proportional to *Sc*^−0.5^ (see Jähne *et al*.^1^), except in the low *Sc* range. It is interesting to recognize that the trends of *k*_L_^+^ against *Sc* are similar between the wind-driven wavy and flat interfaces. This is due to the fact that the mass transfer across the gas-liquid interface is controlled by streamwise vortices on the liquid side even for the wind-driven turbulence under the conditions of low wind velocities without wave breaking (Komori *et al*.^5^). On the other hand, there appear discrepancies in *k*_L_^+^ between the present computations for the wind-driven wavy and flat interfaces and between the present computations and the previous experiments for the wind-driven wavy interfaces. The former discrepancies in *k*_L_^+^ predicted by the present computations between the wind-driven wavy and flat interfaces are considered to be due to the presences of the gravity/capillary waves with Langmuir circulations based on the discussions in Komori *et al*.^5^ and Takagaki *et al*.^6^, in which the mass flux distributions on the gas-liquid interfaces between the wind-driven wavy and flat interfaces are compared. The latter discrepancies in *k*_L_^+^ between the present computations and the previous experiments for the wind-driven wavy interfaces are considered to be due to the presence of surface contamination in the experiments, based on the discussions in Komori *et al*.^4^ and Komori and Shimada^38^. As shown in Fig. 4, the predicted *k*_L_^+^ for the wind-driven wavy interface for *Sc* = 600 is 100~200% larger than the measured values in Iwano *et al*.^17^,^18^ where the free stream wind speed ranges as *U*_∞_ = 3.4~9.5 m/s. Similarly, the present values of *k*_L_^+^ for the wind-driven wavy interface tend to be larger than the measured values in Jähne *et al*.^3^ as a whole. These results agree well with the statement in Komori *et al*.^4^ and Komori and Shimada^38^ that the predicted value of *k*_L_^+^ becomes higher than that in experiments due to the presence of surface contamination in the experiments. It also confirmed that the filtering difference (equation (9)) is negligibly small on present results (e.g. Fig. 4).

**Figure 5:**
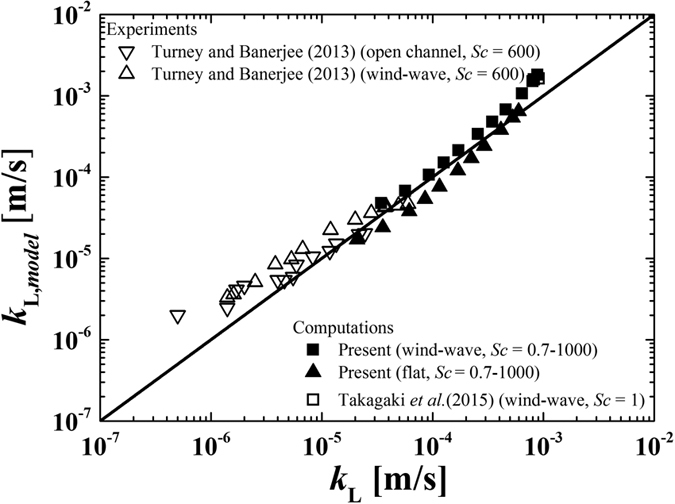
Relationship between measured and modeled values of mass transfer coefficients on both gas and liquid sides . Present values of *k*_L,model_ are estimated using equation (8).

The surface divergence model is considered as one of the models suitable for estimating the gas-liquid mass transfer coefficients (e.g. Banerjee *et al*.^13^, McCready *et al*.^39^). McCready *et al*.^39^ proposed an empirical model for the mass transfer coefficient on the liquid side, *k*_L,model_, as:


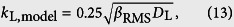


where *D*_L_ is the molecular diffusivity of mass on the liquid side, and *β*_RMS_ is the root-mean-square (RMS) value of the surface divergence, *β*. Here, *β* is defined as:


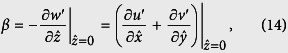


where 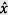 and 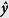 are the tangential directions, and 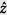 is the normal direction. In addition, *u*′ and *v*′ are the tangential fluctuating velocities in the streamwise and spanwise directions, respectively, and *w*′ is the normal fluctuating velocity. The time-averaged RMS values of *β*_RMS_ are 36.5 s^−1^ (wind-wave) and 4.6 s^−1^ (flat). Figure 5 shows the relationship between mass transfer coefficients, *k*_L_, and the values *k*_L,model_ estimated from equations (13). Here, the vector spacings for calculating the surface divergence are 0.5 mm as in the study by Takagaki *et al*.^6^, and 0.5~0.7 mm and 0.7 mm in the study by Turney and Banerjee^40^ on an open-channel gas-liquid interface, and on a wind-sheared gas-liquid interface, respectively. We can see moderately good agreements between our modeled data and the empirical data of previous studies (Turney and Banerjee^40^). Slight disagreement of equation (13) with experimental and simulated *k*_L_ exists at low and high *Sc*, as seen in Fig. 5, and the coefficient of 0.25 is likely to depend on details of the flow field. Turney and Banerjee^40^ showed that at wind conditions from approximately 4.0 to 10 m/s the capillary waves present in the capillary-gravity mixed wave field will not contribute toward both *k*_L_ due to dynamics of the advection-diffusion equation. This causes the needs for a combination of concepts from the surface renewal model and surface divergence model^40^. For the purposes of this present paper, Fig. 5 shows surface divergence is an approximate proxy for gas-liquid mass transfer in a wide range of Schmidt number (0.7 ≤ *Sc* ≤ 1000).

## Conclusions

In this study, a three-dimensional SEMI-DNS method was applied to a wind-driven turbulence with mass transfer across a sheared wind-driven wavy gas-liquid interface, and the relationship between mass transfer coefficient, *k*_L_, and Schmidt number, *Sc*, was investigated in the wide range of 0.7 ≤ *Sc* ≤ 1000. In order to capture the deforming gas-liquid interface, an arbitrary Lagrangian-Eulerian formulation (ALE) method was employed. The results showed that similar to the cases for flat gas-liquid interfaces, the mass transfer coefficient normalized by friction velocity on the liquid side, *k*_L_^+^ for the wind-driven wavy gas-liquid interface is generally proportional to *Sc*^−0.5^. This trend is endorsed by the fact that the mass transfer across the gas-liquid interface is controlled mainly by streamwise vortices on the liquid side even for the wind-driven turbulence under the conditions of low wind velocities without wave breaking. In addition, the present study showed that *k*_L_ can be roughly estimated by the surface divergence model. For the higher wind velocity conditions, spanwise vortices due to gravity and capillary waves may affect the mass transfer across the interface. The details should be clarified using more powerful supercomputers in a future study.

## Additional Information

**How to cite this article**: Takagaki, N. *et al*. Effect of Schmidt number on mass transfer across a sheared gas-liquid interface in a wind-driven turbulence. *Sci. Rep.* **6**, 37059; doi: 10.1038/srep37059 (2016).

**Publisher's note:** Springer Nature remains neutral with regard to jurisdictional claims in published maps and institutional affiliations.

## Figures and Tables

**Table 1:** Predicted characteristics of gas flow and wind waves.

Case	*t*	*U*_∞_	u*	*U*_10N_	*C*_DN_	*U*_SURF_	*H*_S_	*L*_S_	*C*_P_
	[s]	[m/s]	[m/s]	[m/s]	[−]	[m/s]	[m]	[m]	[m/s]
FLAT	15.0–16.0	4.87	0.241	8.86	7.40 × 10^−4^	0.143	—	—	—
WAVE	5.0–6.0	3.13	0.243	7.21	1.14 × 10^−3^	0.0667	0.00654	0.0488	0.313

*t*: elapsed time, *U*_∞_: free stream wind speed, u*: friction velocity on the gas side, *U*_10N_: wind speed at 10-m height, *C*_DN_: drag coefficient, *U*_SURF_: surface current, *H*_*S*_: significant wave height, *L*_S_: significant wave length, *C*_P_: phase speed of significant wind waves.

## References

[b1] Jähne B., Münnich K. O. & Siegenthaler U. Measurements of gas exchange and momentum transfer in a circular wind-water tunnel. Tellus 31, 321–329 (1979).

[b2] Jähne B. . HE and RN gas exchange experiments in the large wind-wave facility of IMST. J. Geophys. Res. 90, 11989–11998 (1985).

[b3] Jähne B. . On the parameters influencing air-water gas exchange. J. Geophys. Res. 92(C2), 1937–1949 (1987).

[b4] Komori S., Nagaosa R. & Murakami Y. Turbulence structure and mass transfer across a sheared air-water interface in wind-driven turbulence. J. Fluid Mech. 249, 161–183 (1993).

[b5] Komori S. . Direct numerical simulation of wind-driven turbulence and scalar transfer at sheared gas-liquid interfaces. J. Turbulence 11, 1–20 (2010).

[b6] Takagaki N. . Effects of turbulent eddies and Langmuir circulations on scalar transfer in a sheared wind-driven liquid flow. Phys. Fluids 27, 016603 (2015).

[b7] Kurose R., Takagaki N., Kimura A. & Komori S., Direct numerical simulation of turbulent heat transfer across a sheared wind-driven gas-liquid interface, J. Fluid Mech., 804, 646–689, doi: 10.1017/jfm.2016.554 (2016).

[b8] Kunugi T., Satake S. & Ose Y. Direct numerical simulation of carbon-dioxide gas absorption caused by turbulent free surface flow. Int. J. Heat Fluid Flow 22, 245–251 (2001).

[b9] Lakehal D., Fulgosi M., Yadigaroglu G. & Banerjee S. Direct numerical simulation of turbulent heat transfer across a mobile, sheared gas-liquid interface. J. Heat Transfer 125, 1129–1139 (2003).

[b10] Lakehal D., Fulgosi M., Banerjee S. & Yadigaroglu G. Turbulence and heat exchange in condensing vapor-liquid flow. Phys. Fluid 20, 065101 (2008).

[b11] Lakehal D., Fulgosi M. & Yadigaroglu G. Direct numerical simulation of condensing stratified flow. J. Heat Transfer 130, 021501 (2008).

[b12] Banerjee S. Modeling of interphase turbulent transport processes. Ind. Eng. Chem. Res. 46, 3063–3068 (2007).

[b13] Banerjee S., Lakehal D. & Fulgosi M. Surface divergence models for scalar exchange between turbulent streams. Int. J. Multiphase Flow 30, 963–977 (2004).

[b14] Liss P. S. Processes of gas exchange across an air-water interface. Deep-sea Res. 20, 221–238 (1973).

[b15] Broecker H. C., Petermann J. & Siems W. The influence of wind on CO2-exchange in a wind-wave tunnel including the effects of monolayers. J. Marine Res. 36, 595–610 (1978).

[b16] Wanninkhof R. Relationship between wind speed and gas exchange over the ocean. J. Geophys. Res. 97 **(C5**), 7373–7382 (1992).

[b17] Iwano K. . Effect of fetch and entrained bubbles on mass transfer velocity across the wind-driven air-water interface with wave breaking. *In: Proceedings of The 8th KSME-JSME Thermal and Fluids Engineering Conference*, 18–21 May 2012, Songdo Convensia Cener, Songdo, Incheon, Korea (2012).

[b18] Iwano K., Takagaki N., Kurose R. & Komori S. Mass transfer velocity across the breaking air-water interface at extremely high wind speeds. Tellus B 65, 21341 (2013).

[b19] Stolz S. & Adams N. An approximate deconvolution procedure for large-eddy simulation. Phys. Fluids 11, 1699–1701 (1991).

[b20] Komori S. . Direct numerical simulation of three-dimensional open-channel flow with zero-shear gas-liquid interface. Phys. Fluids A5, 115–125 (1993).

[b21] Fulgosi M., Lakehal D., Banerjee S. & De Angelis V. Direct numerical simulation of turbulence in a sheared air-water flow with a deformable interface. J. Fluid Mech. 482, 319–345 (2003).

[b22] Lin M. Y. . Direct numerical simulation of wind-wave generation process. J. Fluid Mech. 616, 1–30 (2008).

[b23] Guo X. & Shen L. Numerical study of the effect of surface wave on turbulence underneath. Part 2. Eulerian and Lagrangian properties of turbulence kinetic energy. J. Fluid Mech. 744, 250–272 (2014).

[b24] Tsai W. T., Chen S. M., Lu G. H. & Garbe C. S. Characteristics of interfacial signatures on a wind-driven gravity-capillary wave. J. Geophys. Res: Oceans 118, 1715–1735 (2013).

[b25] Hasegawa Y. & Kasagi N. Effects of interfacial velocity boundary condition on turbulent mass transfer at high Schmidt numbers. Int. J. Heat Fluid Flow 28, 1192–1203 (2007).

[b26] Hasegawa Y. & Kasagi N. Systematic analysis of high Schmidt number turbulent mass transfer across clean, contaminated and solid interfaces. Int. J. Heat Fluid Flow 29, 765–773 (2008).

[b27] Hasegawa Y. & Kasagi N. Hybrid DNS/LES of high Schmidt number mass transfer across turbulent air-water interface. Int. J. Heat Fluid Flow 52, 1012–1022 (2009).

[b28] Kurose R. . Application of flamelet model to LES of turbulent reacting liquid flows. AIChE J. 57, 911–917 (2011).

[b29] Kurose R. . Subgrid scale scalar variance in high-Schmidt-number turbulence. AIChE J. 58, 377–384 (2012).

[b30] Mathew J., Lechner R., Foysi H. & Friedrich R. An explicit filtering method for LES of compressible flows. Phys. Fluids, 15, 2279–2289 (2003).

[b31] Schwertfirm F. & Manhart M. ADM-modelling aproach for semi-direct numerical simulation of turbulent mixing and mass transport. In: Humphrey J. A. C., Gatski T. B., Eaton J. K., Kasagi N., Friedrich R., Leschziner M. A. (eds.) In Procs. of 4th Int. Symp. on Turbulence and Shear Flow Phenomena, Williamsburg, June 27–29, vol. 2, pp. 823–828 (2005).

[b32] Harlow F. H. & Welch J. E. Numerical calculation of time-dependent viscous incompressible flow of fluid with free surface. Phys. Fluids 8, 2182–2189 (1965).

[b33] Hanratty T. J. Effect of gas flow on physical absorption. Air-Water Gas Transfer ASCE (Civil Engineers) 10–33 (1991).

[b34] Hasegawa Y. & Kasagi N. Turbulent mass transfer across an air-water interface at high Schmidt numbers. Transactions of JSME B 69, 824–832 (2003).

[b35] Hasegawa Y. & Kasagi N. Dependency of local scalar flux on surface divergence at a turbulent air-water interface. Transactions of JSME B 72, 1206–1213 (2006).

[b36] Calmet I. & Magnaudet J. High-Schmidt number mass transfer through turbulent gas-liquid interfaces. Int. J. Heat Fluid Flow 19, 522–532 (1998).

[b37] Merlivat L. & Memery L. Gas exchange across an air-water interface: Experimental results and modeling of bubble contribution to transfer. J. Geophys. Res. 88, 707–724 (1983).

[b38] Komori S. & Shimada T. Gas transfer across a wind-driven air-water interface and the effects of sea water on CO2 transfer. In Air-Water Gas Transfer (Eds. Jahne B. & Monahan E. C.), AEON Verlag & Studio, Hanau, Germany 553–569 (1995).

[b39] McCready M. J., Vassilliadou E. & Hanratty T. J. Computer simulation of turbulent mass transfer at a mobile interface. AIChE J. 32, 1108–1115 (1986).

[b40] Turney D. E. & Banerjee S. Air-water gas transfer and near-surface motions. J. Fluid Mech. 733, 588–624 (2013).

